# Clinical utility of a type 1 diabetes genetic risk score measured as part of MODY genetic testing

**DOI:** 10.2337/dc26-1081

**Published:** 2026-10-01

**Authors:** Jacques Murray Leech, Aparajita Sriram, Kevin Colclough, Andrew T. Hattersley, Jayne A.L. Houghton, Thomas W. Laver, Kashyap A. Patel

**Affiliations:** 1 Institute of Biomedical and Clinical Sciences, University of Exeter, Exeter, UK; 2 Exeter Genomics Laboratory, Royal Devon University Healthcare NHS Foundation Trust, Exeter, UK

## Abstract

**Objective:**

To evaluate the clinical utility of incorporating a type 1 diabetes genetic risk score (T1DGRS) into monogenic diabetes gene panel testing for individuals referred with suspected maturity-onset diabetes of the young (MODY) from routine clinical practice.

**Research Design and Methods:**

We studied 1,129 insulin-treated individuals referred for MODY genetic testing. All underwent gene panel testing including 10 variants to calculate T1DGRS. We compared T1DGRS distributions with reference populations to estimate the T1D prevalence within referrals. We defined age-specific T1DGRS thresholds to identify probable T1D (positive predictive value ≥80%) and applied them in genetically unsolved individuals to assess its utility as a pre-genetic testing triage tool.

**Results:**

T1DGRS in MODY referrals was intermediate between T1D and non-T1D reference populations, consistent with an estimated 20% (95% CI 14.9–25.2) T1D in this real-world setting. After genetic testing, no excess T1DGRS was observed in solved-MODY whereas unsolved-MODY cases showed enrichment consistent with 26.0% T1D, highest in children (76.2%) and lowest in adults (16.8%). Age-specific T1DGRS thresholds identified 16.1% (95% CI 13.8–18.5%) of genetically unsolved as probable T1D. As a pre-testing tool, T1DGRS showed limited discrimination overall (AUC 0.60), but performed well in children under 10years (AUC 0.83), where >50% of tests could be avoided without missing MODY.

**Conclusions:**

Incorporating a T1DGRS into routine MODY gene panel referrals is feasible and clinically valuable. This approach broadens genomic diagnostics beyond monogenic disease by identifying T1D in genetically unsolved cases. Pre-genetic testing utility is age dependent and may reduce unnecessary testing in paediatric patients.

## Introduction

Correctly classifying early-onset diabetes remains a major clinical challenge, particularly when distinguishing maturity-onset diabetes of the young (MODY) from other forms of diabetes. MODY is a monogenic, familial form of diabetes that accounts for approximately 3.6% of diabetes diagnosed before the age of 30 (1). Type 1 diabetes (T1D) is the most common alternative diagnosis in this age group, however, due to overlapping clinical presentation, ~80% of MODY cases are initially misclassified as T1D or type 2 diabetes (T2D)(2). Accurate identification of MODY is important as it enables treatment optimisation, cascade testing, and informed reproductive counselling (3).

Patients referred for MODY genetic testing in routine clinical practice represent a clinically selected group enriched for monogenic diabetes but also include individuals with atypical common diabetes, including individuals with T1D who do not fit the classical autoimmune presentation and therefore prompt consideration of monogenic diabetes. MODY is rare, and testing all young-onset diabetes is neither feasible nor cost-effective. Clinicians therefore prioritise patients for testing. Selection is guided by clinical criteria, expert judgement, and/or the MODY probability calculator (4–6). In insulin-treated individuals, islet autoantibodies and severe insulin deficiency (C-peptide <200 pmol/L, a measure of endogenous insulin secretion) help exclude T1D (5). However, these markers are imperfect. Up to 10% of individuals with T1D are autoantibody negative, and C-peptide is often preserved near diagnosis, with 5–15% retaining persistent secretion post-diagnosis (7,8). Consequently, some insulin-treated individuals referred for MODY testing have atypical T1D. Despite careful selection, ~80% of insulin-treated referrals remain genetically unsolved (no pathogenic variant identified in known MODY genes), leaving many without a definitive diagnosis after genetic testing (9,10). This represents a key unmet clinical need, and identifying atypical T1D in this group resolves diagnostic uncertainty and helps clinicians provide appropriate T1D-directed treatment.

The Type 1 Diabetes Genetic Risk Score (T1DGRS) distinguishes T1D from non-autoimmune diabetes by quantifying an individual’s genetic susceptibility of T1D (11,12). This creates a strong biological contrast with non-autoimmune diabetes such as MODY and has been shown to complement C-peptide and islet autoantibody testing (11,13). A simplified T1DGRS based on the 10 strongest single nucleotide polymorphisms (SNP) provides a cost-effective alternative to larger scores incorporating more loci, supporting feasibility in clinical settings (14). T1DGRS could therefore help identify atypical T1D among individuals referred for MODY testing, particularly in insulin-treated cases where T1D is the most common alternative diagnosis. However, its clinical utility as part of routine MODY genetic testing remains unknown.

In this study, we evaluated the clinical utility of incorporating a 10-SNP T1DGRS into monogenic diabetes gene panel testing for insulin-treated individuals referred with suspected MODY. We used a T1DGRS to quantify T1D within this population and to identify T1D among individuals who remained genetically unsolved after testing. We also assessed whether a T1DGRS could support patient prioritisation for MODY genetic testing by identifying individuals with a higher likelihood of monogenic diabetes.

## Research Design and Methods

### Study cohorts

#### T1D and non-autoimmune reference cohort

We obtained European T1D and non-T1D reference populations from the Wellcome Trust Case Control Consortium (WTCCC), a large population-based genetic study described previously (15). The T1D cohort included individuals diagnosed before age 17 years who required continuous insulin treatment for at least six months (n = 1,963). The non-autoimmune reference group comprised individuals with type 2 diabetes (n = 1,924), an appropriate comparator population given that T1D genetic risk scores show similar distributions in individuals with type 2 diabetes, non-diabetic controls, and those with genetically confirmed MODY (11,12). T2D diagnosis was based on treatment history or laboratory-confirmed hyperglycaemia. We excluded individuals with known MODY or neonatal diabetes.

#### MODY referral cohort

We analysed probands referred from routine clinical care to the Exeter Genomics Laboratory (Royal Devon University Healthcare NHS Foundation Trust, Exeter, UK) for genetic testing due to clinical suspicion of MODY. We included insulin-treated individuals diagnosed before age 40 years with preserved or unknown C-peptide (>200 pmol/L) and negative or unknown islet autoantibody status. These criteria exclude classical autoimmune diabetes while retaining diagnostically uncertain cases in whom MODY and atypical T1D overlap. Referring clinicians measured biomarkers locally. All participants or their guardians provided informed consent. The North Wales Ethics Committee approved the study, and the Genetic Beta Cell Research Bank approved sample access (522/WA/0268). Supplementary Table 1 summarises clinical characteristics at referral.

#### Genetic testing

We performed targeted next-generation sequencing as described by Ellard *et al* (16). Genetic ancestry was inferred using an adapted LASER approach for targeted sequencing (17). Due to the small number of non-European individuals and the lack of appropriate reference cohorts, we restricted our analysis to individuals of non-Finnish European ancestry (n = 1,129), aligning with the reference populations. Variant interpretation and classification followed the American College of Medical Genetics and Genomics/Association of Molecular Pathology criteria, and Association for Clinical Genomic Science best practice guidelines (18,19). MODY referrals were defined based on genetic testing outcome. We defined MODY-solved individuals based on the presence of pathogenic or likely pathogenic variants, with all others classified as MODY-unsolved.

#### T1DGRS calculation

We calculated a T1DGRS using the top 10 SNPs identified by Oram *et al*., as highly discriminative for T1D (11). Briefly, the 10-SNP T1DGRS was represented by the number of risk-increasing alleles at each SNP (0, 1, or 2) multiplied by the SNP-specific weight (natural logarithm of the odds ratio) (Oram et al, Supplementary Table 1) (11). Targeted sequencing captured these variants during routine testing. WTCCC reference samples were genotyped using the Affymetrix 500K SNP array with detailed description of quality control and genotyping described previously (15). As a sensitivity analysis, we constructed a 30-SNP T1DGRS in the reference cohorts, using the same methodology, as described in detail by Oram *et al*. (11). We expressed T1DGRS as percentiles relative to the WTCCC T1D reference distribution based on ranked values in the reference cohort, ranking T1DGRS values within the WTCCC T1D reference population and assigning each participant a percentile based on their position within this distribution. A participant at the 90th percentile therefore has a T1DGRS exceeding 90% of the T1D reference population.

#### Statistical analysis

To estimate the prevalence of T1D within the MODY referral cohort, we employed the “means method” described in detail by Evans *et al*.(20), and extensively used in previous studies (21,22). In brief, the MODY referral cohort was assumed to represent a mixture of autoimmune T1D and non-autoimmune diabetes. The proportion of T1D at the group level was estimated by comparing the mean T1DGRS of the MODY referral cohort with the mean scores of reference T1D and non–autoimmune populations, determining where the MODY referral cohort mean observed relative to the reference means. As a complementary approach, we applied the Earth Mover’s Distance method (20) which uses the full T1DGRS distribution to identify the mixture of reference T1D and non–autoimmune control distributions that minimizes the distance to the observed distribution in the MODY referral cohort. We derived 95% confidence intervals using 2,000 bootstrap resamples. We stratified analyses by genetic testing outcome and age at diagnosis (<10, 10–18, >18 years).

To identify individuals likely to have T1D within the MODY referral cohort, we performed simulation studies to derive empirical T1DGRS thresholds corresponding to a positive predictive value (PPV) of ≥80%. Simulations used WTCCC reference populations of T1D and non–autoimmune diabetes, combined in varying proportions to represent prior T1D prevalences of 20%, 30%, and 75%, spanning the range of values previously observed in age-stratified MODY-unsolved referral groups. These thresholds therefore represent pragmatic clinical decision points that can be adapted according to the prior probability of T1D in different clinical settings. For each simulated mixture, the PPV of T1DGRS was estimated across a range of thresholds, and PPV curves were generated using 1,000 bootstrap resamples of the reference populations. The T1DGRS threshold for each prevalence mixture was defined as the lowest percentile at which the mean PPV first reached ≥80%. These thresholds were then applied to MODY-unsolved individuals according to age at diagnosis. Individuals exceeding the relevant threshold were classified as likely T1D, and their clinical characteristics were compared with the remaining genetically unsolved cohort to assess enrichment for clinical features consistent with a T1D-like phenotype.

Clinical features between groups were compared using the Mann–Whitney U test for continuous variables, including T1DGRS distributions, and Fisher’s exact test for categorical variables. We used logistic regression and receiver operating characteristic (ROC) analysis to evaluate discriminatory performance of the T1DGRS. Optimal T1DGRS percentile thresholds for pre-genetic testing discrimination were derived using Youden’s J statistic.

We performed all analyses in R (version 4.4.1).

## Results

### T1D is common among insulin-treated MODY referrals

**Figure 1:**
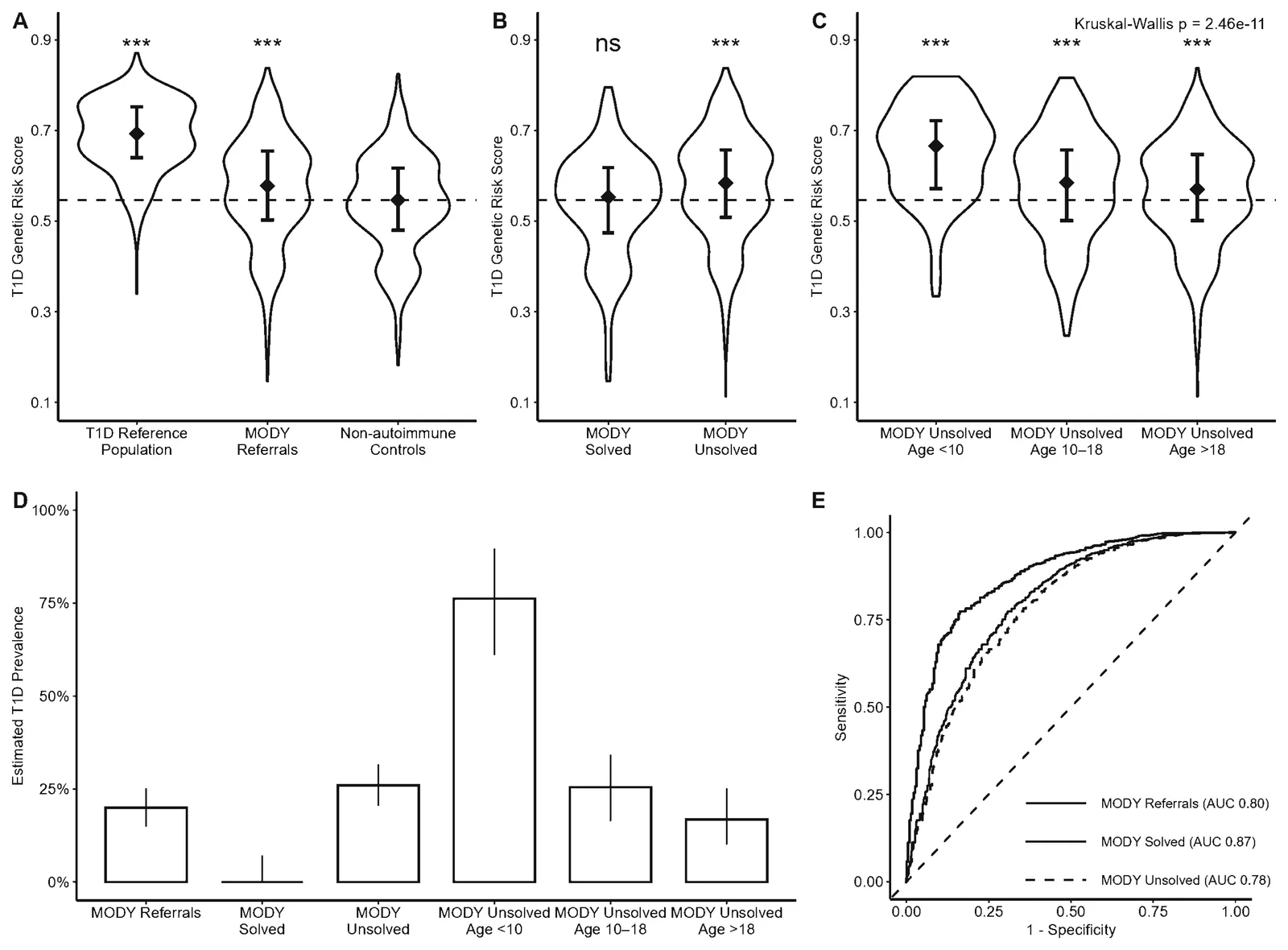
Excess T1D genetic risk in the MODY referral cohort. (A) Distribution of T1DGRS (10 loci) in European individuals referred for MODY testing to the Exeter Genomics Laboratory from routine clinical care who were insulin treated (n = 1,129), compared with reference T1D (n = 1,963) and non–autoimmune diabetes groups (n = 1,924) from the WTCCC. (B) T1DGRS in the MODY referral cohort stratified by genetic testing outcome (MODY-solved, n = 228; MODY-unsolved, n = 901). (C) T1DGRS in MODY-unsolved individuals stratified by age at diagnosis (<10 years, n = 96; 10–18 years, n = 293; >18 years, n = 512). Statistical differences across age categories were assessed using the Kruskal–Wallis test. In (A–C), diamonds indicate medians and error bars indicate interquartile ranges, Statistical significance versus non–autoimmune controls was assessed using the Mann–Whitney U test (*p < 0.05, ***p < 0.00001). (D) Estimated prevalence of T1D in the MODY referral cohort and subgroups, derived using the means method. (E) Receiver operating characteristic curves for T1DGRS distinguishing T1D from MODY. The dashed line indicates no discrimination (AUC = 0.5).

Insulin-treated individuals referred for MODY genetic testing represent a diagnostically challenging group enriched for both monogenic diabetes and T1D with atypical presentations. As genetically confirmed MODY is not associated with an increased autoimmune genetic risk, enrichment of the T1DGRS among the MODY referrals would indicate the presence of misclassified T1D. Among 1,129 European individuals, the median T1DGRS was higher than in the non–autoimmune diabetes reference group (0.58 vs 0.55, p < 1.5 × 10^−14^) and lower than in the T1D reference group (0.69, p < 3 × 10^−171^) (Figure 1A). This intermediate distribution indicates that the referral cohort comprises a mixture of T1D and non-autoimmune diabetes. By modelling the T1DGRS distribution against reference populations, we estimated that 20.0% (95% CI 14.9–25.2%) of insulin-treated referrals had atypical T1D (Supplementary Table 2).

Genetic testing identified MODY causing variants in 20.2% of referrals (n = 228), with the remaining 901 individuals genetically unsolved. The individuals with a genetic diagnosis of MODY showed no excess T1D genetic risk compared with non–autoimmune diabetes controls (median difference 0.006, p = 0.88). In contrast, the genetically unsolved MODY referrals showed higher T1DGRS (median difference 0.04, p = 6.8 × 10^−19^) (Figure 1B), corresponding to an estimated 26.0% (95% CI 20.5–31.7%) prevalence of atypical T1D (Supplementary Table 2, Figure 1D).

We next assessed how the atypical T1D estimate in the genetically unsolved MODY group varied by age at diagnosis (<10, 10–18, >18 years). The T1DGRS was highest in individuals diagnosed at less than age 10 years and was lowest in those diagnosed above age 18 years (median difference 0.09, p < 2.2 × 10^−12^). In line with this, the estimated prevalence was highest in children diagnosed before age 10 years (76.2%, 95% CI 61.0–89.7%), lower in those diagnosed at 10–18 years (25.5%, 95% CI 16.4–34.2%), and lowest in adults (>18 years: 16.8%, 95% CI 10.1–25.2%) (Figure 1C–D). A sensitivity analysis using the Earth Mover’s Distance approach produced similar estimates (Supplementary Table 2).

Finally, we assess discriminatory performance of T1DGRS in out cohort. Discrimination using T1DGRS was high when comparing individuals with a genetic diagnosis of MODY with reference T1D cases (AUC 0.87, 95% CI 0.85–0.90). Discrimination fell in the unsolved MODY group (AUC 0.78, 95% CI 0.76–0.80), consistent with a higher prevalence of T1D in this subgroup (Figure 1E). As this referral cohort reflects routine clinical practice, biochemical data were not uniformly available due to variation in testing. Sensitivity analyses showed no difference in T1DGRS between individuals with complete (n = 854) and incomplete (n = 271) C-peptide or autoantibody data (median difference 0.007, p = 0.67), suggesting that missing data did not materially affect results among 1,129 individuals (Supplementary Figure 1).

### T1DGRS enables post–genetic test identification of individuals with probable T1D

Having identified age-dependent enrichment of individuals with likely T1D among genetically unresolved MODY referrals, we next assessed whether the T1DGRS could identify these individuals at the individual level. Empirically derived T1DGRS thresholds corresponding to a high probability of T1D (PPV ≥ 80%) were established for children, adolescents, and adults (2%, 56%, and 76%, respectively, Supplementary Figure 2), enabling classification of unsolved referrals as likely T1D cases. This reclassification identified 16.1% of unsolved MODY individuals (n = 145, 95% CI = 124–167) as probable T1D cases (95% CI 13.8–18.5%), capturing approximately 61.9% of the estimated T1D cases in this group based on the means method estimate.

To validate these findings, we compared clinical characteristics of the probable T1D group with the remaining genetically unsolved individuals (n = 756). The reclassified group showed a distinct phenotype, with younger age at diagnosis, shorter diabetes duration, lower BMI, and lower C-peptide levels (all p < 0.05, Table 1). A sensitivity analysis using percentile cutoffs generated from the 30-SNP T1DGRS yielded similar thresholds (Supplementary Figure 1), demonstrating consistency of classification across different T1DGRS versions.

### Age-dependent utility of T1DGRS as a pre-genetic testing screening tool

**Figure 2:**
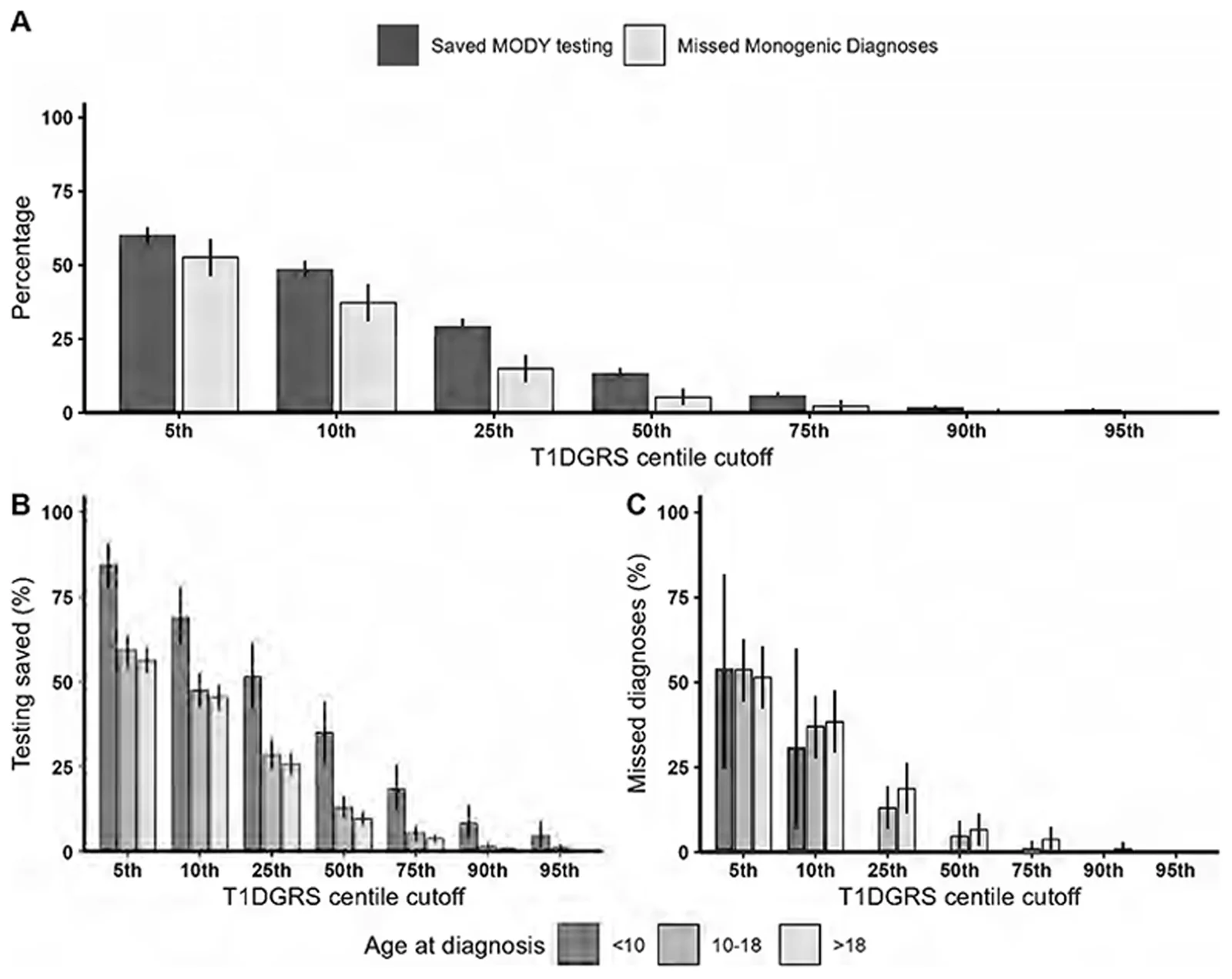
Age-dependent utility of T1DGRS thresholds for pre-genetic testing screening in MODY referrals. (A) Proportion of MODY genetic tests avoided (“testing saved”) and proportion of genetically confirmed MODY cases missed across increasing T1DGRS percentiles of the T1D reference distribution in the full referral cohort. Darker bars indicate testing saved, and lighter bars indicate missed diagnoses. (B) Proportion of testing saved stratified by age at diagnosis (<10, 10–18, >18 years). (C) Proportion of genetically confirmed MODY cases missed stratified by age at diagnosis. In (B–C), darker bars indicate younger age groups and lighter bars indicate older age groups. Error bars represent 95% confidence intervals.

We assessed the performance of T1DGRS as a pre-genetic testing screening tool to identify individuals with a low likelihood of monogenic diabetes and reduce unnecessary genetic testing. In the overall cohort, the T1DGRS showed limited discrimination between genetically confirmed MODY and non-monogenic cases (AUC 0.601, 95% CI 0.561–0.642) (Supplementary Figure 3). We assessed the impact of excluding individuals based on different T1DGRS thresholds prior to genetic testing, quantifying both the reduction in testing and the number of missed genetically confirmed MODY cases. Across the cohort, lower thresholds reduced the number of individuals undergoing genetic testing but increased the proportion of missed MODY diagnosis (Figure 2A). For example, restricting testing to individuals with T1DGRS below the 5th percentile of the T1D reference distribution would exclude 676 individuals (59.9%) from testing but miss 120 MODY genetic diagnosis (52.6%). Using the optimal threshold defined by Youden’s J statistic (<22.6th percentile) would have removed 31.1% cases but would have missed 16.2% confirmed MODY cases (Supplementary Table 3). In this clinically selected population, avoiding any missed diagnoses would require an extremely high threshold (95th percentile), excluding fewer than 1% of samples.

Given the age-dependent prevalence of T1D, we stratified analyses by age group. In adolescents and adults, the T1DGRS showed similarly limited discrimination (AUC 0.61 and 0.55 respectively) (Supplementary Figure 4). In contrast, performance was higher in children under 10 years (AUC 0.83, 95% CI 0.73–0.91), with a more favourable balance between reducing testing and maintaining diagnostic sensitivity (Figure 2B–C). At the optimal threshold (22.8th percentile), no MODY cases were missed in this subgroup, while 53.2% (95% CI 43.9–62.3%) of individuals could have been excluded from testing (Supplementary Table 3). These findings suggest that, although the T1DGRS is not suitable as a universal pre-screening tool, its improved performance in children may support further evaluation of its use in age-specific MODY referral pathways.

## Discussion

In this large study of T1DGRS applied within routine clinical genetic testing for MODY referrals, we estimate that approximately 20% of individuals had atypical T1D. T1DGRS can help identify these cases after genetic testing, reducing diagnostic uncertainty. However, as a pre-screening tool in genomic laboratories, T1DGRS showed limited value in adolescents and adults, but clear potential to reduce unnecessary genetic testing in young children.

Our study provides the first estimate of atypical T1D among clinically referred individuals with suspected MODY, highlighting the need for improved tools to support diagnosis in this setting. By using a mixture modelling approach independent of predefined thresholds, we generated a real-world estimate of T1D prevalence within routine MODY referrals. One in five individuals had T1D at the point of genetic testing, despite prior clinical selection designed to exclude clear cases of autoimmune diabetes (4–6). This prevalence varied by age and remained consistent across analytical approaches, with no excess T1D signal observed in genetically confirmed MODY cases, supporting the robustness of our findings. These results reveal a significant diagnostic gap that could be bridged through improved stratification strategies, such as the integration of genetic and emerging biomarkers (12,23,24).

The most immediate clinical value of T1DGRS lies in resolving the large proportion of individuals who remain genetically unsolved after comprehensive testing. In our cohort, 79.8% of patients had no identified monogenic cause, leaving substantial uncertainty in disease classification and management. Probability-based T1DGRS thresholds enabled individual-level classification, identifying 16.1% of this genetically unresolved MODY referrals as having a high probability of T1D. This reclassification is supported by clear phenotypic differences, including younger age at diagnosis, lower BMI, and reduced C-peptide, consistent with T1DGRS identifying a distinct subgroup of individuals with features of atypical T1D. Assigning a probable T1D classification reduces diagnostic uncertainty, supports appropriate management, and may limit unnecessary further genetic testing. Notably, this approach extends the role of genomic laboratories beyond binary outputs by enabling a clinically meaningful, diagnostic classification within the genetically unsolved MODY group. Although this has the potential to influence clinical care, its impact on clinical management and patient outcomes will require prospective evaluation in routine practice.

Translating T1DGRS from a research tool into a clinical report requires careful consideration. Unlike monogenic testing, which provides a binary result, T1DGRS generates a continuous measure of genetic risk that may be less intuitive for clinicians and patients (25). A probability-based framework, supported by likelihood ratios and age-specific risk estimates, would improve interpretability and clinical utility (26). Reports should include clear guidance for non-specialists, outlining how a “probable T1D” result informs classification, management, and the need for further investigation (25,27). Standardised reporting will be essential to ensure consistency in assay calibration, thresholds, and interpretation across laboratories (27,28). Importantly, a high T1DGRS should not be presented as a definitive diagnosis but as a strong probabilistic indicator that supports clinical decision-making within the broader diagnostic pathway.

We evaluated T1DGRS at the point of genetic testing, where patients have already undergone clinical selection for suspected MODY and therefore represent an atypical, diagnostically challenging population. In this context, discrimination is inherently lower than in classical case–control settings, as comparisons occur between overlapping phenotypes rather than distinct disease groups (11). This reduction in performance is expected and emphasises the importance of interpreting T1DGRS within the clinical pathway. Within this setting, the utility of T1DGRS as a pre-genetic testing triage tool was strongly age dependent. In children, where autoimmune diabetes predominates, T1DGRS discriminated well (AUC 0.83), and the optimal threshold (22.7th percentile) captured all MODY cases in this cohort while potentially avoiding 53.2% of tests. In contrast, in adolescents and adults, no threshold allowed exclusion of MODY without missing some genetically confirmed cases. This suggests that improved diagnostic strategies are needed in these older age groups, where a substantial proportion of MODY diagnoses are made, to account for the increasing prevalence of non-autoimmune diabetes. In resource-limited settings, T1DGRS may still provide a pragmatic, low-cost triage tool despite a small risk of missed cases. For example, using a threshold of 25% could reduce testing by 29.1% while missing only 14.9% of genetically confirmed MODY cases. However, as MODY is a clinically actionable diagnosis with implications for treatment and family screening, stricter thresholds are difficult to justify in well-resourced settings. T1DGRS should therefore be applied flexibly, according to the priorities and constraints of the healthcare system (3).

Despite the prospective clinical setting, which enhances real-world applicability, our study has limitations. Biochemical investigations were performed by referring clinicians at local hospitals as part of routine clinical care rather than within a central study laboratory. Consequently, assay platforms and diagnostic performance characteristics (including sensitivity and specificity) for islet autoantibody testing could not be standardised or consistently reported across centres, limiting comparability of biomarker data across sites within this real-world clinical pathway. Although a small proportion of individuals had missing biochemical data, sensitivity analyses showed similar T1DGRS distributions between those with complete and incomplete data, suggesting minimal impact on the findings. The analysis was restricted to individuals of European ancestry, which may limit generalisability to other populations, such as South Asians, who have a higher prevalence of lean, young-onset type 2 diabetes (29). This restriction was necessary to match the WTCCC reference cohorts and represents a methodological strength (15). While the T1DGRS has been validated in several non-European populations, further studies are needed to determine whether the clinical utility observed in this MODY referral pathway extends to more diverse ancestry groups (30). We used the 10-SNP T1DGRS due to its cost-effectiveness and limited incremental gains from additional variants, however, newer scores such as T1DGRS2 and T1DGRS-MAPS may further improve discrimination (31,32). As genomic sequencing becomes more widely implemented, incorporating enhanced genetic risk models could refine classification.

In summary, incorporating T1DGRS into MODY genetic testing extends its clinical utility beyond monogenic diagnosis. It enables identification of T1D among genetically unsolved referrals, providing diagnostic clarity for patients and clinicians. In children, T1DGRS may also reduce unnecessary genetic testing while maintaining diagnostic accuracy.

## Supplementary Material

Supplementary Data

## Figures and Tables

**Table 1:** Clinical characteristics of unsolved MODY individuals with probable T1D versus other genetically unsolved MODY patients Values for continuous variables are presented as median (interquartile range), and categorical variables as count (percentage). Continuous variables were compared using the Wilcoxon rank-sum test, and categorical variables using Fisher’s exact test. BMI = Body Mass Index.

Characteristics	Unsolved MODY – Probable T1D	Other Unsolved MODY	P Value
N	145	756	NA
Age Diabetes Diagnosis, years	9 (5, 15)	23 (16, 30)	1.2×10^-33^
Sex (female)	73 (48.1%)	439 (58.6%)	0.04
BMI, kg/m^2^	22.7 (20.2, 27.0)	27.5 (23.8, 31.6)	1.3×10^-14^
HbA1c, %	7.4 (6.2, 9.2)	8.4 (6.7, 10.0)	1.2×10^-3^
Diabetes Duration, years	3.2 (1.2, 9.0)	8.0 (1.8, 16.9)	6.4×10^-6^
C-peptide, pmol/L	532.8 (306.0, 960.5)	720 (444.0, 1130.0)	3.4×10^-3^
Parental Diabetes	59 (42.4%)	486 (65.3%)	6.01×10^-7^

## Data Availability

Data from the MODY cohort is not publicly available due to patient confidentiality and ethical restrictions. Access may be granted to approved researchers via the Genetic Beta Cell Research Bank (https://www.diabetesgenes.org/current-research/genetic-beta-cell-research-bank/), subject to steering committee approval. Contact the corresponding author for details.

## Article Highlights

- Why did we undertake this study?
  Distinguishing MODY from atypical type 1 diabetes remains a clinical challenge, often leading to misdiagnosis and unnecessary, expensive genetic testing.
- What is the specific question(s) we wanted to answer?
  Can a type 1 diabetes genetic risk score (T1DGRS) identify hidden type 1 diabetes in MODY referrals and optimize the clinical testing pipeline?
- What did we find?
  Approximately 20% of referrals were hidden type 1 diabetes cases. The T1DGRS reclassified 16% of unsolved cases and demonstrated the potential to reduce genetic testing burden in children.
- What are the implications of our findings?
  Integrating the T1DGRS into MODY testing pathways can offer diagnostic clarity for patients and clinicians after negative monogenic testing and has the potential to reduce testing burden in children.
