# Supplementary material for: Clinical utility of a type 1 diabetes genetic risk score measured as part of MODY genetic testing
Source: Diabetes Care. Author manuscript; Available in PMC 2026 Oct 10. (PMC7619526; doi:10.2337/dc26-1081)
Supplement: Supplementary file 1 [file EMS217145-supplement-Supplementary_Data.docx]

**Supplementary Tables and Figures**

| **Characteristics** | **MODY-Solved** | **MODY-Unsolved** |
| --- | --- | --- |
| N | 228 | 901 |
| Age Diabetes Diagnosis, years | 18 (14, 26) | 21 (14, 29) |
| Sex (female) | 152 (66.6%) | 512 (56.8%) |
| BMI, kg/m² | 24.7 (21.6, 27.8) | 26.8 (23.0, 31.0) |
| HbA1c, % | 7.7 (6.6, 9.7) | 8.7 (6.9, 10.7) |
| Diabetes Duration, years | 5.7 (1.5, 18.9) | 6.2 (1.6, 15.4) |
| C-peptide, pmol/L | 700 (431, 1081) | 670 (400, 1078) |
| Parental Diabetes | 163 (73.4%) | 45 (61.7%) |

**Supplementary Table 1. Clinical characteristics of genetically solved vs genetically unsolved MODY referrals**

Values for continuous variables are presented as median (interquartile range), and categorical variables as count (percentage). Continuous variables were compared using the Wilcoxon rank-sum test, and categorical variables using Fisher’s exact test. BMI = Body Mass Index.

| **Group** | **Method** | **T1D Prevalence Estimate (95 CI)** | **T1D N estimate (95% CI)** |
| --- | --- | --- | --- |
| All | Means Method | 0.200 (0.149, 0.252) | 226 (169, 284) |
| MODY Solved | Means Method | 0.000 (0.000, 0.071) | 0 (0,16) |
| MODY Unsolved | Means Method | 0.260 (0.205, 0.317) | 234 (185, 286) |
| Mody Unsolved  Age <10 | Means Method | 0.762 (0.610, 0.897) | 73 (59,86) |
| Mody Unsolved  Age 10–18 | Means Method | 0.255 (0.164, 0.342) | 75 (48,100) |
| Mody Unsolved  Age >18 | Means Method | 0.168 (0.101, 0.252) | 86 (52, 123) |
| All | Earth Mover’s Distance | 0.201 (0.158, 0.249) | 227 (175, 281) |
| MODY Solved | Earth Mover’s Distance | 0.000 (0.000, 0.115) | 0 (0, 26) |
| MODY Unsolved | Earth Mover’s Distance | 0.263 (0.206, 0.316) | 236 (185, 284) |
| Mody Solved  Age <10 | Earth Mover’s Distance | 0.762 (0.622, 0.899) | 73 (59, 86) |
| Mody Solved  Age 10–18 | Earth Mover’s Distance | 0.255 (0.167, 0.346) | 75 (49 ,101) |
| Mody Solved  Age >18 | Earth Mover’s Distance | 0.173 (0.106, 0.242) | 88 (54,124) |

**Supplementary Table 2. Estimated prevalence of type 1 diabetes among individuals referred for MODY testing**

Prevalence of T1D was estimated using two methods, means method and Earth Mover’s Distance. Estimates are presented both as proportions and as estimated numbers of individuals, with corresponding 95% confidence intervals. Subgroups are defined by genetic testing result (MODY solved vs MODY unsolved) and, for MODY-unsolved individuals, by age at diagnosis (<10, 10–18, >18 years).

| **Age Group** | **Percentile** | **Sensitivity** | **Specificity** | **J Index** | **Testing Saved (%)** | **Diagnosis**  **Missed (%)** |
| --- | --- | --- | --- | --- | --- | --- |
| All | 5 | 0.474 | 0.618 | 0.092 | 59.97 | 52.63 |
| All | 10 | 0.627 | 0.512 | 0.139 | 48.36 | 37.28 |
| All | 22.6* | 0.838 | 0.349 | 0.186 | 31.09 | 16.23 |
| All | 25 | 0.851 | 0.327 | 0.178 | 29.14 | 14.91 |
| All | 50 | 0.947 | 0.152 | 0.099 | 13.20 | 5.26 |
| All | 75 | 0.978 | 0.065 | 0.044 | 5.67 | 2.19 |
| All | 90 | 0.996 | 0.02 | 0.016 | 1.68 | 0.44 |
| All | 95 | 1 | 0.011 | 0.011 | 0.89 | 0.00 |
| <10 | 5 | 0.462 | 0.885 | 0.347 | 84.40 | 53.85 |
| <10 | 10 | 0.692 | 0.74 | 0.432 | 68.81 | 30.77 |
| <10 | 22.8* | 1 | 0.604 | 0.604 | 53.21 | 0 |
| <10 | 25 | 1 | 0.583 | 0.583 | 51.38 | 0 |
| <10 | 50 | 1 | 0.396 | 0.396 | 34.86 | 0 |
| <10 | 75 | 1 | 0.208 | 0.208 | 18.35 | 0 |
| <10 | 90 | 1 | 0.094 | 0.094 | 8.26 | 0 |
| <10 | 95 | 1 | 0.052 | 0.052 | 4.59 | 0 |
| 10-18 | 5 | 0.463 | 0.611 | 0.074 | 59.10 | 53.70 |
| 10-18 | 10 | 0.63 | 0.512 | 0.142 | 47.38 | 37.04 |
| 10-18 | 24.9* | 0.87 | 0.341 | 0.212 | 28.43 | 12.96 |
| 10-18 | 25 | 0.87 | 0.341 | 0.212 | 28.43 | 12.96 |
| 10-18 | 50 | 0.954 | 0.16 | 0.114 | 12.97 | 4.63 |
| 10-18 | 75 | 0.991 | 0.068 | 0.059 | 5.24 | 0.93 |
| 10-18 | 90 | 1 | 0.02 | 0.02 | 1.5 | 0 |
| 10-18 | 95 | 1 | 0.014 | 0.014 | 1 | 0 |
| >18 | 5 | 0.486 | 0.572 | 0.058 | 56.22 | 51.40 |
| >18 | 10 | 0.617 | 0.469 | 0.086 | 45.40 | 38.32 |
| >18 | 18.8* | 0.766 | 0.352 | 0.118 | 33.12 | 23.36 |
| >18 | 25 | 0.813 | 0.271 | 0.085 | 25.69 | 18.69 |
| >18 | 50 | 0.935 | 0.102 | 0.036 | 9.53 | 6.54 |
| >18 | 75 | 0.963 | 0.037 | 0 | 3.72 | 3.74 |
| >18 | 90 | 0.991 | 0.006 | -0.003 | 0.65 | 0.94 |
| >18 | 95 | 1 | 0.002 | 0.002 | 0.16 | 0 |

**Supplementary Table 3. Diagnostic performance of the T1DGRS as a pre-screening tool for monogenic diabetes testing.**

Performance of the T1DGRS in discriminating MODY-solved individuals from genetically unsolved referrals prior to monogenic diabetes testing. Performance is stratified by age group and shown across standard T1DGRS percentiles and the optimal threshold (asterisk) based on maximum Youden’s J-index.

**Supplementary Figure 1: T1DGRS distribution according to data completeness**

Distribution of the 10-loci T1D genetic risk score (T1DGRS) in European individuals referred for MODY testing, stratified by data completeness: complete C-peptide (CP) and autoantibody data (n = 854) versus partial missing data (n = 271; missing either autoantibody testing or CP measurement). Individuals missing both CP and autoantibody data (n = 4) were excluded from this analysis due to the small sample size.

**Supplementary Figure 2: Positive Predictive Value Curves of T1DGRS across varying T1D prevalences**

PPV curves were generated using the T1D genetic risk score to distinguish type 1 diabetes cases from non-automimune diabetes controls. Curves correspond to three assumed population prevalences of T1D: 0.2 (solid-black), 0.3 (solid-grey), and 0.8 (dashed-black). PPV was calculated as the proportion of individuals above each T1DGRS percentile who were true T1D cases. Horizontal dashed lines indicate a PPV of 0.8. Each curve represents the average PPV across 1000 bootstrap resamples of 1000 individuals sampled according to the specified prevalence

**Supplementary Figure 3: Receiver Operating Characteristic Curve (ROC) to discriminate MODY-solved against MODY-unsolved cases**

ROC curve illustrating the performance of the T1DGRS in distinguishing individuals with a pathogenic MODY variant (MODY-solved, n = 228) from those without a causative variant (MODY-unsolved, n = 901), with an area under the curve (AUC) of 0.601 (95% CI: 0.561–0.642). The grey dashed line represents an AUC of 0.5, corresponding to no discriminative ability.

**Supplementary Figure 4: Receiver Operating Characteristic Curve to discriminate MODY-solved against MODY-unsolved cases stratified by age group**

ROC curve illustrating the performance of the T1DGRS in distinguishing individuals with a pathogenic MODY variant from those without a causative variant, stratified by age. The solid black line represents children aged <10 years (N=96), achieving an AUC of 0.826 (95% CI: 0.725–0.912). The solid grey line indicates adolescents aged 10–18 years (N=293), with an AUC of 0.607 (95% CI: 0.549–0.668), while the dashed black line represents adults aged >18 years (N=512), with an AUC of 0.55 (95% CI: 0.496–0.608). The diagonal light grey dashed line represents an AUC of 0.5, signifying no discriminative ability.
